# Periostin: A Downstream Mediator of EphB4-Induced Osteogenic Differentiation of Human Bone Marrow-Derived Mesenchymal Stem Cells

**DOI:** 10.1155/2016/7241829

**Published:** 2015-12-16

**Authors:** Fei Zhang, Zehua Zhang, Dong Sun, Shiwu Dong, Jianzhong Xu, Fei Dai

**Affiliations:** ^1^National & Regional United Engineering Laboratory of Tissue Engineering, Third Military Medical University, Chongqing 400038, China; ^2^Department of Orthopaedics, Southwest Hospital, Third Military Medical University, Chongqing 400038, China; ^3^Department of Biomedical Materials Science, School of Biomedical Engineering, Third Military Medical University, Chongqing 400038, China

## Abstract

Erythropoietin-producing hepatocyte B4 (EphB4) has been reported to be a key molecular switch in the regulation of bone homeostasis, but the underlying mechanism remains poorly understood. In this study, we investigated the role of EphB4 in regulating the expression of periostin (POSTN) within bone marrow-derived mesenchymal stem cells (MSCs) and assessed its effect and molecular mechanism of osteogenic induction in vitro. Treatment with ephrinB2-FC significantly increased the expression of POSTN in MSCs, and the inhibition of EphB4 could abrogate this effect. In addition, osteogenic markers were upregulated especially in MSCs overexpressing EphB4. To elucidate the underlying mechanism of cross talk between EphB4 and the Wnt pathway, we detected the change in protein expression of phosphorylated-glycogen synthase kinase 3*β*-serine 9 (p-GSK-3*β*-Ser9) and *β*-catenin, as well as the osteogenic markers Runx2 and COL1. The results showed that GSK-3*β* activation and osteogenic marker expression levels were downregulated by ephrinB2-FC treatment, but these effects were inhibited by blocking integrin *α*v*β*3 in MSCs. Our findings demonstrate that EphB4 can promote osteogenic differentiation of MSCs via upregulation of POSTN expression. It not only helps to reveal the interaction mechanism between EphB4 and Wnt pathway but also brings a better understanding of EphB4/ephrinB2 signaling in bone homeostasis.

## 1. Introduction 

Osteogenic differentiation of bone marrow-derived mesenchymal stem cells (MSCs) is essential for normal bone homeostasis and the repair of bone defects. Thus, the molecules that have osteoinduction activity play a critical role in these processes. Among them, erythropoietin-producing hepatocyte B4 (EphB4) and its ligand ephrinB2 have attracted much interest for use in regulating bone homeostasis [1, 2]. Bone homeostasis depends on a delicate balance between bone formation and bone absorption. The coordinated function of osteoblasts and osteoclasts ensures the normal metabolic processes of bone. As a receptor, expression of EphB4 in MSCs/preosteoblasts can promote osteogenic differentiation through forward signaling upon binding to ephrinB2, which is expressed in osteoclasts in response to cell-cell contact, and reverse signaling through ephrinB2 also can inhibit the osteoclastogenesis [3]. Thus, EphB4/ephrinB2 interaction is known to be involved in the balance of bone formation and adsorption in bone homeostasis; however, the exact molecular mechanism remains poorly understood.

In addition to osteoinductive molecules, the local microenvironment also plays critical roles in the regulation of the bone homeostasis and repair. Extracellular matrix (ECM) is an important part of the microenvironment and is vital to the regulation of cell adhesion, migration, and differentiation via cell-ECM interactions. In bone, the ECM is composed primarily of several molecular complexes of proteins, such as collagen (CO), laminin (LM), osteopontin (OP), fibronectin (FN), osteonectin (ON), and other glycoproteins [4, 5]. A recent study also showed that periostin (POSTN), which is also referred to as osteoblast-specific factor 2 (OSF-2), is present in bone ECM and plays a crucial role in cell-ECM interactions [6]. POSTN is a matricellular protein that has been shown to regulate the survival and differentiation of osteoblasts through binding integrin *α*v*β*3 and regulating Wnt/*β*-catenin signaling [7]. It also is preferentially expressed by periosteal osteoblasts in response to stimulation with parathyroid hormone (PTH), transforming growth factor-beta (TGF-*β*), and bone morphogenetic protein-2 (BMP-2) as well as mechanical stimulation [7–10]. Based on these findings, we hypothesized that POSTN may be a downstream mediator involved in EphB4-induced osteogenic differentiation of MSCs. To test the hypothesis, we systematically investigated the osteogenic function of POSTN and the relationship between EphB4, POSTN, and Wnt/*β*-catenin signaling during osteogenesis* in vitro*.

## 2. Materials and Methods

### 2.1. Isolation, Expansion, and Identification of MSCs

MSCs were isolated and cultured using previously described methods [11]. Briefly, approximately 20 mL of iliac crest marrow aspirate was obtained from healthy adult donors at the Southwest Hospital, and Institutional Review Board-approved informed consent was obtained in accordance with the Declaration of Helsinki. The marrow samples were diluted with 20 mL phosphate-buffered saline (PBS) and added over 20 mL Percoll solution (Pharmacia Corporation, USA) with a density of 1.073 g/mL in a 50 mL conical tube. Then, nucleated cells were separated by density gradient centrifugation (900 ×g for 30 min at 20°C). Cells were resuspended and cultured at a density of 7.5 × 10^6^ cells per 37.5 cm^2^ flask in human MSC basal medium containing 10% fetal bovine serum (OriCell Human Mesenchymal Stem Cell Growth Medium, Cyagen Biosciences, Guangzhou, China) at 37°C in 5% CO_2_. When cells reached 90% confluence, they were detached using 0.25% trypsin/EDTA (HyClone, Logan, UT, USA) and replated at a density of 1.8 × 10^5^ cells per 37.5 cm^2^ flask. UltraCULTURE Serum-Free Medium (Lonza, Allendale, NJ, USA) was used when necessary.

Cells were harvested at the third passage for identification of surface antigens by flow cytometry. About 5 × 10^5^ cells were incubated with a phycoerythrin- (PE-) labeled monoclonal antibody to CD34, CD45, CD73, CD90, or CD166 or fluorescein isothiocyanate- (FITC-) labeled monoclonal antibody to CD105 (all from BD Biosciences, San Jose, CA, USA) for 30 min in room temperature. Cells were washed and resuspended in PBS, and data were acquired on a FACS Calibur (BD Biosciences). MSCs were confirmed based on the expression of CD73, CD90, CD105, and CD166 and the lack of CD34 and CD45 expression.

### 2.2. Immunohistochemistry (ICH)

SABC IHC kits (Zhongshan Corporation, Beijing, China) were used for immunohistochemical staining with a primary antibody to POSTN (Abcam, Cambridge, MA, USA), and nuclei were counterstained with hematoxylin. Images were captured using a Leica Microsystem microscope (DFC300 FX; Heerbrugg, Switzerland).

### 2.3. Infection of MSCs with Adenovirus Containing the EphB4 Gene and Small Interfering RNA (siRNA) Sequences

MSCs were infected with recombinant adenoviruses expressing the EphB4 gene or siRNA sequences at a multiplicity of infection(MOI) of 40 for 72 h as previously described [12]. The efficiency of infection was assessed by western blotting. The EphB4 siRNA sequences were as follows: forward: 5′-GUACUAAGGUCUACAUCGAdTdT-3′ and reverse: 5′-UCGAUGUAGACCUUAGUACTdTd-3′.

### 2.4. Functions of EphB4 and POSTN in Osteogenic Differentiation

EphrinB2-FC (4 *μ*g/mL, R&D Systems, Minneapolis, MN, USA) and POSTN (500 ng/mL, R&D Systems) were added individually to promote osteogenic differentiation. NVP-BHG-712 (BHG712, 50 ng/mL, R&D Systems) was used as a EphB4 kinase inhibitor. Integrin *α*v*β*3 on MSCs was blocked by incubation with a mouse monoclonal antibody (2 *μ*g/mL, Abcam) at 37°C for 2 h before exposure to ephrinB2-FC or POSTN as a control treatment.

The culture medium was replaced with special conditional osteogenic differentiation medium (OriCell MSC Osteogenic Differentiation Medium, Cyagen Biosciences), which was then refreshed every 3 days. Alkaline phosphatase (ALP) staining was performed with a FAST BCIP/NBT tablet (Sigma-Aldrich, St. Louis, MO, USA) after 9 days in culture, and calcium nodules were stained by 0.4% Alizarin red S staining (Sigma-Aldrich) after 21 days in culture.

### 2.5. Real-Time Polymerase Chain Reaction (PCR)

Total RNA was extracted using the QIAGEN Rneasy Mini kit (QIAGEN, Hilden, Germany) and quantified using an ultraviolet spectrophotometer (Beckman Coulter DU-600, Indianapolis, IN, USA). Then, cDNA was synthesized from 4 *μ*g total RNA using the PrimeScript 1st Strand cDNA Synthesis Kit (TaKaRa, Shiga, Japan), and real-time quantitative PCR was carried out using the 7500 qPCR System (Applied Biosystems, Foster City, CA, USA) with SYBR Premix EX Taq II (TaKaRa). Glyceraldehyde-3-phosphate dehydrogenase (GAPDH) was used for reference.

The primer sequences used were as follows:

 POSTN: forward primer, GCCATCACATCGGACATA, and reverse primer, CTCCCATAATAGACTCAGAACA.
 Runx2: forward primer, AGATGATGACACTGCCACCTCTG, and reverse primer, GGGATGAAATGCTTGGGAACTGC.
 Osterix: forward primer, ACTGGCTAGGTGGTGGTCAG, and reverse primer, GGTAGGGAGCTGGGTTAAGG.
 
*α*-SMA: forward primer, CATGGCATCATCACCAACTG, and reverse primer, GCTGGGACATTGAAAGTCTC.
 ALP: forward primer, ACCATTCCCACGTCTTCACATTTG, and reverse primer, AGACATTCTCTCGTTCACCGCC.
 COL1: forward primer, CCTGGAAAGAATGGAGATGATG, and reverse primer, ATCCAAACCACTGAAACCTCTG.
 GAPDH: forward primer, ACCCATCACCATCTTCCAGGAG, and reverse primer, GAAGGGGCGGAGATGATGAC.

### 2.6. Western Blot Analysis

Cells were starved for 12 h, and then the medium was replaced with fresh serum-free medium. Antibodies to ephrinB2-Fc, POSTN, NVP-BHG-712, and integrin *α*v*β*3 were added as previously described. After 5 days, total protein was prepared in radioimmunoprecipitation (RIPA) lysis buffer (KeyGEN BioTECH, Beijing, China) and quantified by bicinchoninic acid assay (Pierce Chemical, Dallas, TX, USA). Then, 20 *μ*g protein was separated by 8% sodium dodecyl sulfate- (SDS-) polyacrylamide gel electrophoresis (PAGE) and transferred onto polyvinylidene fluoride (PVDF) membranes (Millipore, Billerica, MA, USA). Membranes were blocked in 3% bovine serum albumin (BSA) in Tris-buffered saline (TBS) and incubated overnight at 4°C with primary antibodies against POSTN, COL1 (both 1 : 1500, Abcam), GSK-3*α*/*β*, phosphorylated-GSK-3*α*/*β* (Ser21/9), *β*-catenin, Runx2 (all 1 : 1000, Cell Signaling Technology, Danvers, MA, USA), and GAPDH (1 : 12000, Sanjian, Tianjin, China). Membranes were washed with PBS containing 0.1% Triton X-100 and incubated with horseradish peroxidase- (HRP-) anti-rabbit IgG or anti-mouse IgG (Amersham Biosciences/GE Healthcare, Amersham, UK) at a dilution of 1 : 5000 as a secondary antibody for 1 h at room temperature. The signals on the membranes were visualized by ChemiDoc XRS (Bio-Rad, Hercules, CA, USA) with an enhanced chemiluminescence kit (Amersham Biosciences) and quantitatively analyzed using Image J2x software (National Institutes of Health, Bethesda, MD, USA).

### 2.7. Enzyme-Linked Immunosorbent Assay (ELISA)

POSTN concentrations in serum-free medium collected from MSC which were treated by ephrinB2-Fc or BHG712 as previously described were measured using the Human Periostin ELISA Kit (RayBiotech, Norcross, GA, USA) according to the manufacturer's instructions.

The concentrations of phosphorylated EphB4 were measured using the Human Phospho-EphB4 DuoSet IC ELISA kit (R&D Systems) according to the manufacturer's instructions and as previously described [13].

### 2.8. Statistical Analysis

Data are expressed as mean ± standard deviation (SD) values. Statistical analysis was conducted with two-way analysis of variance (ANOVA) using the GraphPad Prism 5.0 statistical software package (GraphPad, La Jolla, CA, USA). *P* < 0.05 was considered statistically significant.

## 3. Results

### 3.1. MSC Isolation and POSTN Expression

**Figure 1:**
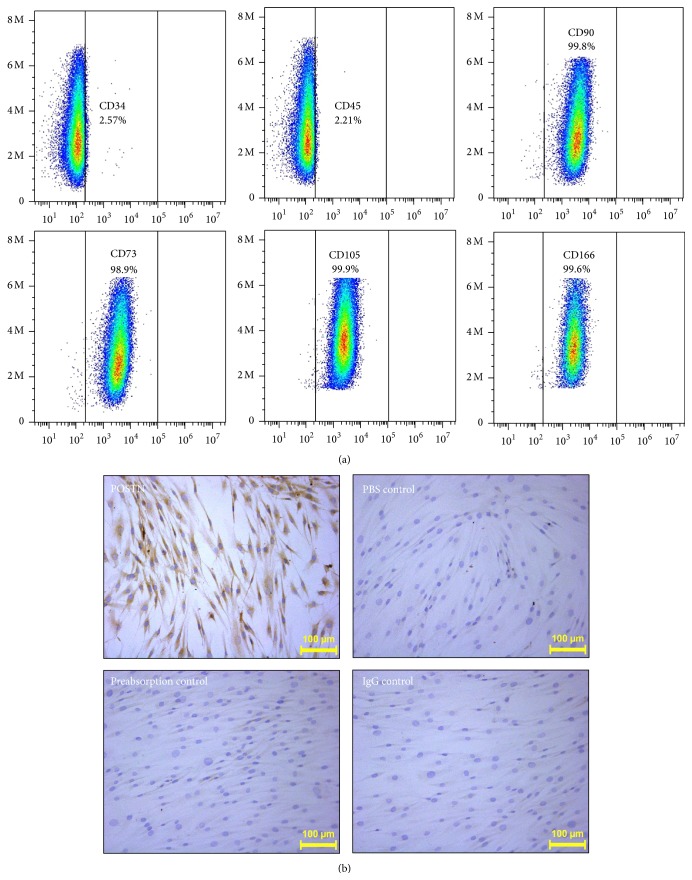
MSC isolation and evaluation of POSTN expression. (a) The purity of MSCs was verified by FACS according to positive staining for CD73, CD90, CD105, and CD166 and negative staining for CD34 and CD45. (b) The level and localization of POSTN expression within MSCs were detected by immunohistochemistry (magnification, ×100).

FACS analysis demonstrated expression of CD73, CD90, CD105, and CD166 and a lack of expression of CD34 and CD45 in third passage MSCs, indicating the purity and successful expansion of MSCs* in vitro *(Figure 1(a)). After the adherence of MSCs to cover slips, POSTN expression was detected by immunohistochemical staining. PBS or IgG instead of the primary antibody and preabsorption of the primary antibody were used as negative controls (Figure 1(b)). Cytoplasmic staining of POSTN was observed in all MSCs.

### 3.2. Upregulation of POSTN Expression Induced by EphB4

**Figure 2:**
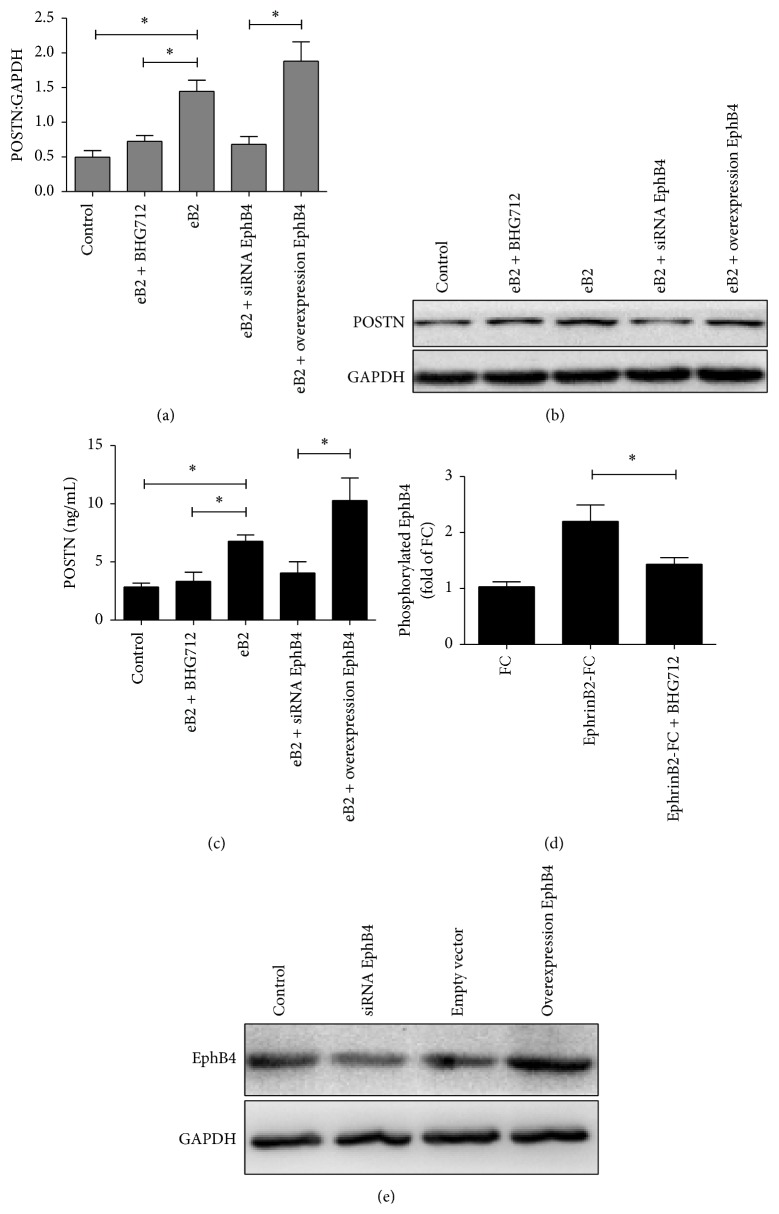
Activation of EphB4 increased POSTN expression in MSCs. (a) POSTN mRNA levels were quantified by real-time PCR upon stimulation with ephrinB2-FC (eB2) for 72 h in serum-free medium. Untreated MSCs were used as the control. Bars represent means ± SD from 3 biological replicates relative to GAPDH controls; ^*∗*^ *P* < 0.05. (b) Protein expression of POSTN was detected using western blotting under the same conditions. (c) The concentration of POSTN in the serum-free medium was assessed by ELISA. Bars represent means ± SD from 3 biological replicates; ^*∗*^ *P* < 0.05. (d) The level of phosphorylated EphB4 was detected by ELISA using starved MSCs after stimulation with ephrinB2-Fc (4 *μ*g/mL) for 20 minutes. Fc was used as the control. (e) The efficiency of siRNA and overexpression of EphB4 were evaluated by western blot analysis. MSCs alone were used as the control.

To observe POSTN expression induced by EphB4 signaling, we detected POSTN expression at the protein and mRNA levels using western blotting and real-time PCR, respectively, as well as POSTN concentrations in serum-free medium after stimulation of MSCs with ephrinB2-FC. The data showed that POSTN expression was increased significantly upon stimulation with ephrinB2-FC only in wild-type MSCs and MSCs overexpressing EphB4 but not in the BHG712- and EphB4 siRNA-treated groups (Figures 2(a) and 2(b)). In addition, POSTN concentrations in serum-free medium were assessed by ELISA, and similar results were obtained (Figure 2(c)).

**Figure 3:**
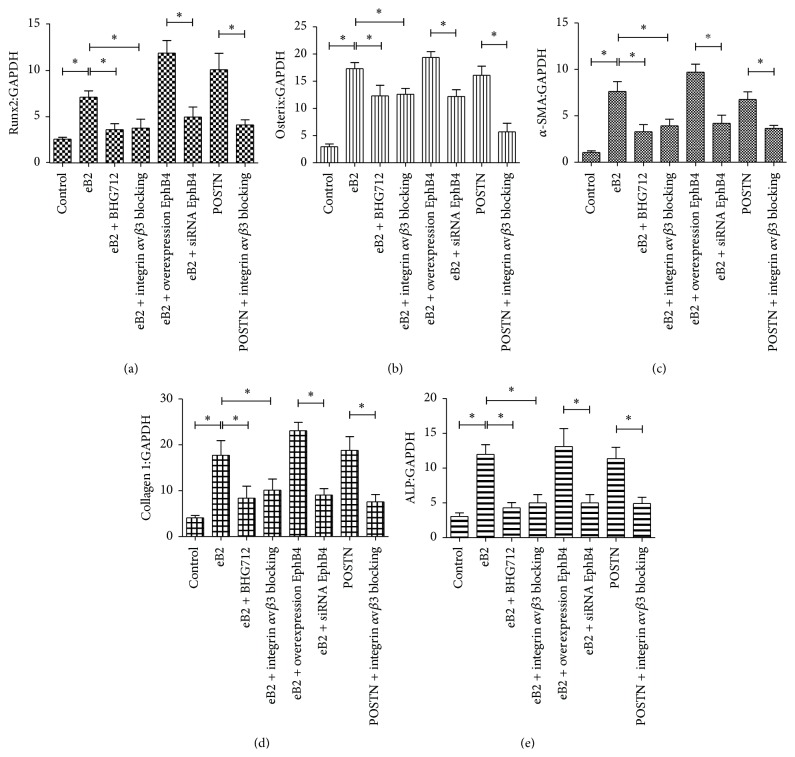
mRNA expression of osteoblast markers after treatment with ephrinB2-FC or POSTN. (a~e) mRNA expression was quantified by real-time PCR upon stimulation with ephrinB2-FC or POSTN for 3 (Runx2, Osterix), 6 (*α*-SMA, COL1), and 9 (ALP) days. Untreated MSCs were used as the control. Bars represent means ± SD from 3 biological replicates relative to GAPDH controls; ^*∗*^ *P* < 0.05.

First, the function of BHG 712 in suppressing EphB4 phosphorylation was tested by ELISA (Figure 2(d)), and the efficiency of siRNA and overexpression of EphB4 were confirmed by western blot analysis (Figure 2(e)). Then, to evaluate the osteogenic induction potential of ephrinB2-FC through increased POSTN expression, expression of the osteogenic markers Runx2, Osterix, *α*-SMA, COL1, and ALP was quantified by real-time PCR after stimulation of MSCs with ephrinB2-FC. MSCs in which POSTN expression was stimulated and in which integrin *α*v*β*3 was blocked were used as positive and negative controls, respectively. The data showed that, upon stimulation with ephrinB2-FC, the expression of osteogenic markers increased significantly only in wild-type MSCs and MSCs overexpressing EphB4 but not in the BHG712-treated, integrin *α*v*β*3-blocked, and EphB4 siRNA-treated groups of MSCs (Figure 3). These results suggest that POSTN expression can be upregulated by activation of EphB4 and that upregulation of POSTN promotes the osteogenic differentiation of MSCs.

### 3.3. Confirmation of EphB4-Induced Osteogenic Differentiation via POSTN

**Figure 4:**
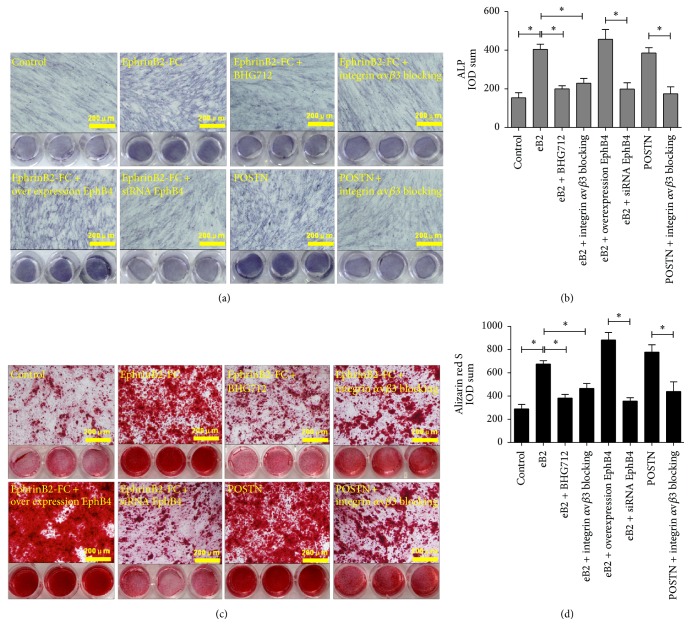
Evaluation of osteogenic differentiation after treatment with ephrinB2-FC or POSTN. (a) ALP staining was performed after 9 days in culture in osteogenic medium with stimulation. Untreated MSCs were used as the control. (b) The sum integral optical density (IOD) was quantified using Image-Pro Plus 6.0. Bars represent means ± SD from 3 biological replicates; ^*∗*^ *P* < 0.05. (c) Alizarin red S staining was done after 21 days in culture in the same conditions. (d) The sum IOD again was quantified by Image-Pro Plus 6.0. Bars represent means ± SD from 3 biological replicates; ^*∗*^ *P* < 0.05.

To assess the osteogenic differentiation of MSCs upon stimulation with ephrinB2-FC or POSTN, advanced osteogenesis markers of ALP production and bone nodule formation were detected via specialized staining in 24-well plates. ALP staining was assessed after 9 days in culture under stimulation with ephrinB2-FC or POSTN in osteogenic medium, and quantification of the sum integral optical density (IOD) was performed. The data showed that, with stimulation by ephrinB2-FC, the sum IOD of ALP staining was increased significantly in wild-type MSCs and MSCs overexpressing EphB4. However, this phenomenon was not observed in the BHG712-treated, integrin *α*v*β*3-blocked, and EphB4 siRNA-treated groups. MSCs in which POSTN expression was stimulated and in which integrin *α*v*β*3 was blocked were used as positive and negative controls, respectively. The sum IOD of ALP was also increased in POSTN-treated MSCs but not in MSCs after blockage of integrin *α*v*β*3 (Figures 4(a) and 4(b)). The sum IOD of Alizarin red S staining followed the same trends as that for ALP staining (Figures 4(c) and 4(d)). These data showed that both ephrinB2-FC and POSNT can induce the osteogenic differentiation of MSCs. However, blockage of integrin *α*v*β*3, which is the specific receptor of POSTN, resulted in inhibition of the osteogenic induction ability of ephrinB2-FC, suggesting that POSTN is as a downstream mediator of EphB4-induced osteogenic differentiation among MSCs.

### 3.4. Mechanism of EphB4-Induced Osteogenic Differentiation

**Figure 5:**
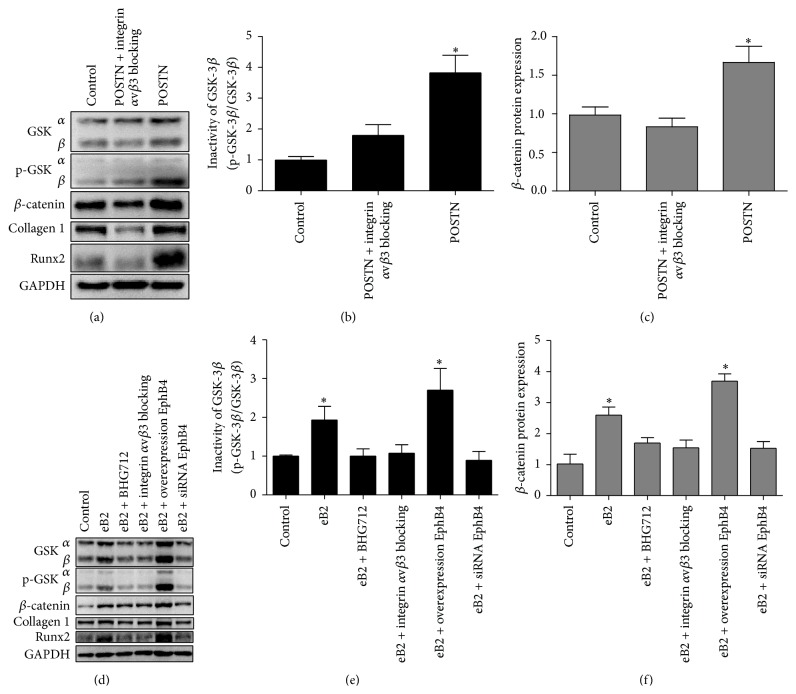
Osteogenic differentiation mechanism of the EphB4 pathway. (a) POSTN treatment and integrin *α*v*β*3 blocking were applied to MSCs for 3 days, and cell lysates (20 *μ*g each) were subjected to western blotting. Untreated MSCs were used as the control. (b, c) The levels of inactive GSK (p-GSK-3*β*-Ser9/GSK-3*β*) and *β*-catenin were quantified using ImageJ2x and expressed as a fold-change over that of the control. Bars represent means ± SD from 3 biological replicates; ^*∗*^ *P* < 0.05. (d) Integrin *α*v*β*3 blocking, ephrinB2-FC treatment, and BHG712 treatment were applied for 3 days, and cell lysates (20 *μ*g each) were extracted for western blotting. Again, untreated MSCs were used as the control. (e, f) The levels of inactive GSK (p-GSK-3*β*-Ser9/GSK-3*β*) and *β*-catenin were quantified using ImageJ2x and expressed as a fold-change over that of the control. Bars represent means ± SD from 3 biological replicates; ^*∗*^ *P* < 0.05.

The osteogenic differentiation effect of POSTN was detected by western blotting. We cultured integrin *α*v*β*3-blocked and POSTN-treated MSCs in serum-free medium for 3 days, and the level of p-GSK-3*β*-Ser9 was significantly greater in the POSTN-treated group than in the other groups. In addition, the expression of *β*-catenin and other osteogenesis markers, including Runx2 and COL1, also was greater in POSTN-treated MSCs (Figure 5(a)). Measurement of inactive GSK-3*β* (p-GSK-3*β*-Ser9/GSK-3*β*) and *β*-catenin showed that POSTN could decrease the amount of active GSK-3*β* and increase *β*-catenin expression significantly, and this effect was not observed in MSCs after blockage of integrin *α*v*β*3 (Figures 4(b) and 4(c)). These data suggest that POSTN can promote osteogenic differentiation through the Wnt pathway by downregulating active GSK-3*β*.

To determine the osteogenic mechanism of EphB4 signaling, we cultured ephrinB2-FC-treated cells in serum-free medium for 3 days, and the BHG712-treated, integrin *α*v*β*3-blocked, EphB4 siRNA-treated, and EphB4 overexpressing groups of MSCs were prepared as described above. The data showed that activation of EphB4 also could increase the level of p-GSK-3*β*-Ser9 to downregulate the level of active GSK-3*β* except in the BHG712-treated and EphB4 siRNA-treated groups, and the expression of *β*-catenin, Runx2, and COL1 followed the same trend as p-GSK-3*β*-Ser9 (Figures 5(d)~5(f)). Moreover, blockage of integrin *α*v*β*3 in MSCs also could suppress the downregulation of active GSK-3*β* induced by the activation of EphB4. These results imply that an increase in POSTN expression induced by EphB4 signaling may be responsible for the cross talk with the Wnt pathway in promoting the osteogenic differentiation of MSCs.

## 4. Discussion

Elucidation of the coupling mechanism in bone homeostasis is important in promoting the research for treatment of bone defects and related diseases. The discovery of EphB4/ephrinB2 in regulating osteogenesis is helpful for explaining the coupling mechanism, but the downstream mechanism has not been fully elucidated. Our results show that the activation of EphB4 upregulates the expression of POSTN, and this may help to explain the cross talk between EphB4 and the Wnt pathway in promoting the osteogenic differentiation of MSCs.

Many studies have verified that the bone ECM and the corresponding cell-ECM reaction are crucial for bone remodeling and homeostasis through regulation of cell adhesion, migration, and differentiation [14–17]. The function of POSTN as an ECM protein in bone formation has been determined recently. POSTN is a secreted protein that is highly expressed in MSCs/preosteoblasts and supports cell adhesion, spreading, and differentiation [18]. In addition, expression of integrin *α*v*β*3, the receptor of POSTN, has been observed in MSCs/preosteoblasts [19, 20]. Some studies have indicated that POSTN contributes to the development of bone mass and maintenance of bone strength. Gerbaix et al. [21] found that POSTN-deficient mice have low bone mass, and POSTN is essential for the cortical bone response to mechanical forces. Bonnet et al. [22] also found that the recovery of fatigued bones in POSTN-deficient mice is diminished compared to that in wild-type mice, indicating that the level of POSTN expression is important for the healing of bone fatigue fractures. In addition, POSTN was shown to have osteogenic induction activity [6]. Heo et al. [23] found that delivery of recombinant POSTN to human adipose tissue-derived MSCs embedded within a hydroxyapatite/tricalcium phosphate scaffold can accelerate healing of calvarial defects. Oshima et al. [24] showed that POSTN expression is regulated by Twist, a basic helix-loop-helix (bHLH) transcription factor that plays an important role in early osteogenesis. Litvin et al. [25] confirmed that the POSTN isoforms can increase the mRNA expression of osteogenic markers such as Cbfa1 (Runx2), ALP, COL1, osteocalcin, and osteopontin in MC3T3-E1 osteoblast-like cells, suggesting a role for POSTN in osteogenic differentiation. However, the role of POSTN in human MSCs has been rarely reported. Our data systematically show that osteogenic markers such as Runx2, COL1, ALP, and calcium nodule formation were upregulated with an increase in POSTN expression achieved by various experimental methods. Moreover, the osteogenic inductive effect of POSTN was inhibited completely by blocking integrin *α*v*β*3, which confirms the osteogenic inductive effect of POSTN in human MSCs.

Eph (erythropoietin-producing hepatocyte) receptors, the most numerous subfamily of receptor tyrosine kinases (RTKs), and their ephrin ligands are known to be involved in cell-cell communication responsible for the regulation of cell proliferation and differentiation as well as cancer progression [26–28]. Ephs are type I transmembrane allosteric enzymes that include a conserved intracellular tyrosine kinase domain and a unique extracellular ligand binding domain. According to sequence conservation, the Ephs and their ligands have been divided into two subclasses named A and B [29–31]. Although the regulatory effects of EphB4/ephrinB2 in osteogenesis have been reported by many studies, the underlying molecule mechanism has not been fully elucidated [1, 3, 32]. Recently, increasing evidence shows that ephrinB2 expressed in MSCs can be regulated by parathyroid hormone (PTH) and plays a vital role in promoting osteogenic differentiation by preventing osteoblast apoptosis and increasing expression of the osteoblast markers [2, 33–35]. However, the mechanism by which EphB4 induces osteogenic differentiation is incompletely understood, and knowledge of this specific mechanism will be helpful to understand the coupling involved in bone remodeling. In the present study, we found for the first time that POSTN expression is increased upon stimulation with ephrinB2-FC. POSTN was reported to mediate the stimulation of Wnt-*β*-catenin signaling by parathyroid hormone (PTH) [7]. Our results showed that the treatment of POSTN also can increase the expression of *β*-catenin by suppressing the activation of GSK-3*β*. Combined, these findings indicate the possibility of cross talk between EphB4 and the Wnt pathway through upregulation of POSTN expression to promote osteogenic differentiation of MSCs. We know that the Wnt signaling pathway is vital for promoting osteogenic differentiation of MSCs, and that it can suppress chondrogenic and adipogenic differentiation among these cells [36–38]. *β*-catenin, which is a key factor in the canonical Wnt signaling pathway, is also known to be crucial for the osteogenic differentiation of MSCs. As a downstream signaling molecule of GSK-3*β*, *β*-catenin is ubiquitinated and degraded upon phosphorylation of active GSK-3*β* [39], and the phosphorylation level of serine 9 (Ser9) within GSK-3*β* reduces the activation of GSK-3*β* [40, 41]. Our data show that treatment with ephrinB2-FC can suppress activation of GSK-3*β* by increasing the level of p-GSK-3*β*-Ser9 in wild-type MSCs as well as MSCs overexpressing EphB4, and the expression of *β*-catenin and osteogenic markers were affected accordingly. However, this effect was not observed in MSCs treated with EphB4 siRNA or BHG712, which functions as an EphB4 kinase inhibitor [42]. These results imply that EphB4 forward signaling can interact with the Wnt pathway to promote osteogenic differentiation of human MSCs. Moreover, the p-GSK-3*β*-Ser9 level in MSCs after blocking integrin *α*v*β*3 also was deceased, as was the expression of *β*-catenin and osteogenic markers. These findings suggest that the cross talk between EphB4/ephrinB2 signaling and the Wnt pathway is mediated by POSTN.

It should be noted that this study only concentrated on the effect of upregulation of POSTN expression induced by EphB4 signaling with cell-based systems* in vitro*, and the exact mechanism by which activation of EphB4 increases POSTN expression was not determined. In addition, further studies are needed to determine the specific mechanism by which POSTN affects the Wnt pathway in promoting osteogenic differentiation and the osteogenic inductive effect of EphB4-POSTN should be investigated* in vivo* for greater biological relevance. However, our study provides a platform for exploring the molecular osteogenic mechanism of EphB4/ephrinB2 signaling.

In conclusion, we showed that the activation of EphB4 in MSCs treated with ephrinB2-FC can increase the expression of POSTN, and POSTN can promote osteogenic differentiation through cross talk with the Wnt pathway. This is the first report to unravel the relationship between EphB4 signaling, POSTN expression, and the Wnt pathway, and our results provide a novel perspective for understanding the osteogenic mechanism of EphB4/ephrinB2 signaling in human MSCs.

## References

[1] Zhao C., Irie N., Takada Y. (2006). Bidirectional ephrinB2-EphB4 signaling controls bone homeostasis. *Cell Metabolism*.

[2] Takyar F. M., Tonna S., Ho P. W. M. (2013). EphrinB2/EphB4 inhibition in the osteoblast lineage modifies the anabolic response to parathyroid hormone. *Journal of Bone and Mineral Research*.

[3] Matsuo K., Irie N. (2008). Osteoclast–osteoblast communication. *Archives of Biochemistry and Biophysics*.

[4] Ivaska J., Heino J. (2000). Adhesion receptors and cell invasion: mechanisms of integrin-guided degradation of extracellular matrix. *Cellular and Molecular Life Sciences*.

[5] Anselme K. (2000). Osteoblast adhesion on biomaterials. *Biomaterials*.

[6] Merle B., Garnero P. (2012). The multiple facets of periostin in bone metabolism. *Osteoporosis International*.

[7] Bonnet N., Conway S. J., Ferrari S. L. (2012). Regulation of beta catenin signaling and parathyroid hormone anabolic effects in bone by the matricellular protein periostin. *Proceedings of the National Academy of Sciences of the United States of America*.

[8] Horiuchi K., Amizuka N., Takeshita S. (1999). Identification and characterization of a novel protein, periostin, with restricted expression to periosteum and periodontal ligament and increased expression by transforming growth factor *β*. *Journal of Bone and Mineral Research*.

[9] Wen W., Chau E., Jackson-Boeters L., Elliott C., Daley T. D., Hamilton D. W. (2010). TGF-*β*1 and FAK regulate periostin expression in PDL fibroblasts. *Journal of Dental Research*.

[10] Inai K., Norris R. A., Hoffman S., Markwald R. R., Sugi Y. (2008). BMP-2 induces cell migration and periostin expression during atrioventricular valvulogenesis. *Developmental Biology*.

[11] Jaiswal N., Haynesworth S. E., Caplan A. I., Bruder S. P. (1997). Osteogenic differentiation of purified, culture-expanded human mesenchymal stem cells in vitro. *Journal of Cellular Biochemistry*.

[12] Zhang F., Zhang Z., Sun D., Dong S. W., Xu J., Dai F. (2015). EphB4 promotes osteogenesis of CTLA4-modified bone marrow-derived mesenchymal stem cells through cross talk with Wnt pathway in xenotransplantation. *Tissue Engineering Part A*.

[13] Pennisi A., Ling W., Li X. (2009). The ephrinB2/EphB4 axis is dysregulated in osteoprogenitors from myeloma patients and its activation affects myeloma bone disease and tumor growth. *Blood*.

[14] Brunner M., Jurdic P., Tuckerman J. P., Block M. R., Bouvard D. (2013). New insights into adhesion signaling in bone formation. *International Review of Cell and Molecular Biology*.

[15] Hidalgo-Bastida L. A., Cartmell S. H. (2010). Mesenchymal stem cells, osteoblasts and extracellular matrix proteins: enhancing cell adhesion and differentiation for bone tissue engineering. *Tissue Engineering—Part B: Reviews*.

[16] Datta N., Holtorf H. L., Sikavitsas V. I., Jansen J. A., Mikos A. G. (2005). Effect of bone extracellular matrix synthesized in vitro on the osteoblastic differentiation of marrow stromal cells. *Biomaterials*.

[17] Damsky C. H. (1999). Extracellular matrix-integrin interactions in osteoblast function and tissue remodeling. *Bone*.

[18] Galli C., Piergianni M., Piemontese M. (2013). Periostin improves cell adhesion to implantable biomaterials and osteoblastic differentiation on implant titanium surfaces in a topography-dependent fashion. *Journal of Biomedical Materials Research—Part A*.

[19] Su J. L., Chiou J., Tang C. H. (2010). CYR61 regulates BMP-2-dependent osteoblast differentiation through the {alpha}v{beta}3 integrin/integrin-linked kinase/ERK pathway. *The Journal of Biological Chemistry*.

[20] Park Y. S., Hwang S., Jin Y. M. (2015). CCN1 secreted by tonsil-derived mesenchymal stem cells promotes endothelial cell angiogenesis via integrin *α*
_v_
*β*
_3_ and AMPK. *Journal of Cellular Physiology*.

[21] Gerbaix M., Vico L., Ferrari S. L., Bonnet N. (2015). Periostin expression contributes to cortical bone loss during unloading. *Bone*.

[22] Bonnet N., Gineyts E., Ammann P., Conway S. J., Garnero P., Ferrari S. (2013). Periostin deficiency increases bone damage and impairs injury response to fatigue loading in adult mice. *PLoS ONE*.

[23] Heo S. C., Shin W. C., Lee M. J. (2015). Periostin accelerates bone healing mediated by human mesenchymal stem cell-embedded hydroxyapatite/tricalcium phosphate scaffold. *PLOS ONE*.

[24] Oshima A., Tanabe H., Yan T., Lowe G. N., Glackin C. A., Kudo A. (2002). A novel mechanism for the regulation of osteoblast differentiation: transcription of periostin, a member of the fasciclin I family, is regulated by the bHLH transcription factor, Twist. *Journal of Cellular Biochemistry*.

[25] Litvin J., Selim A.-H., Montgomery M. O. (2004). Expression and function of periostin-isoforms in bone. *Journal of Cellular Biochemistry*.

[26] Mertens-Walker I., Fernandini B. C., Maharaj M. S. (2015). The tumour-promoting receptor tyrosine kinase, EphB4, regulates expression of Integrin-*β*8 in prostate cancer cells. *BMC Cancer*.

[27] Li M., Zhao J., Qiao J., Song C., Zhao Z. (2014). EphB4 regulates the growth and migration of pancreatic cancer cells. *Tumor Biology*.

[28] Ferguson B. D., Liu R., Rolle C. E. (2013). The EphB4 receptor tyrosine kinase promotes lung cancer growth: a potential novel therapeutic target. *PLoS ONE*.

[29] Coulthard M. G., Morgan M., Woodruff T. M. (2012). Eph/ephrin signaling in injury and inflammation. *The American Journal of Pathology*.

[30] Himanen J. P. (2012). Ectodomain structures of Eph receptors. *Seminars in Cell and Developmental Biology*.

[31] Janes P. W., Nievergall E., Lackmann M. (2012). Concepts and consequences of Eph receptor clustering. *Seminars in Cell and Developmental Biology*.

[32] Martin T. J., Allan E. H., Ho P. W. M. (2009). Communication between ephrinB2 and EphB4 within the osteoblast lineage. *Osteoimmunology*.

[33] Tonna S., Takyar F. M., Vrahnas C. (2014). EphrinB2 signaling in osteoblasts promotes bone mineralization by preventing apoptosis. *The FASEB Journal*.

[34] Tierney E. G., McSorley K., Hastings C. L. (2013). High levels of ephrinB2 over-expression increases the osteogenic differentiation of human mesenchymal stem cells and promotes enhanced cell mediated mineralisation in a polyethyleneimine-ephrinB2 gene-activated matrix. *Journal of Controlled Release*.

[35] Allan E. H., Häusler K. D., Wei T. (2008). EphrinB2 regulation by PTH and PTHrP revealed by molecular profiling in differentiating osteoblasts. *Journal of Bone and Mineral Research*.

[36] Day T. F., Guo X., Garrett-Beal L., Yang Y. (2005). Wnt/*β*-catenin signaling in mesenchymal progenitors controls osteoblast and chondrocyte differentiation during vertebrate skeletogenesis. *Developmental Cell*.

[37] Hill T. P., Später D., Taketo M. M., Birchmeier W., Hartmann C. (2005). Canonical Wnt/beta-catenin signaling prevents osteoblasts from differentiating into chondrocytes. *Developmental Cell*.

[38] Kennell J. A., MacDougald O. A. (2005). Wnt signaling inhibits adipogenesis through *β*-catenin-dependent and -independent mechanisms. *Journal of Biological Chemistry*.

[39] Baron R., Rawadi G. (2007). Minireview: targeting the Wnt/*β*-catenin pathway to regulate bone formation in the adult skeleton. *Endocrinology*.

[40] Harwood A. J. (2001). Regulation of GSK-3: a cellular multiprocessor. *Cell*.

[41] Baron R., Kneissel M. (2013). WNT signaling in bone homeostasis and disease: from human mutations to treatments. *Nature Medicine*.

[42] Martiny-Baron G., Holzer P., Billy E. (2010). The small molecule specific EphB4 kinase inhibitor NVP-BHG712 inhibits VEGF driven angiogenesis. *Angiogenesis*.

